# Supplementary material for: MetaRibo-Seq measures translation in microbiomes
Source: Nat Commun. 2020 Jun 29;11:3268. doi: 10.1038/s41467-020-17081-z (PMC7324362; doi:10.1038/s41467-020-17081-z)
Supplement: Supplementary file 10 — Supplementary Data 7 [file 41467_2020_17081_MOESM10_ESM.zip › File2/Confidence_VeryHigh_Taxonomy/293402_out.krona.html]

Javascript must be enabled to view this page.

members
magnitude
magnitudeUnassigned
count
unassigned
taxon
rank

293402\_out

29

29
superkingdom
2

29
976
phylum

29
class
200643

order
171549
29

29
family
815

genus
816
29

29

SRS015190\_contig\_number\_1377SRS015431\_contig\_number\_31246SRS016018\_contig\_number\_7049SRS016989\_contig\_number\_14635SRS019068\_contig\_number\_26229SRS019267\_contig\_number\_3330SRS019601\_contig\_number\_13038SRS019787\_contig\_number\_9949SRS020622\_contig\_number\_86SRS024075\_contig\_number\_1539SRS024132\_contig\_number\_contig-100\_427.244536SRS024331\_contig\_number\_contig-100\_467.180659SRS043411\_contig\_number\_3279SRS048870\_contig\_number\_20593SRS048981\_contig\_number\_634SRS053214\_contig\_number\_3026SRS058770\_contig\_number\_570SRS075878\_contig\_number\_18106SRS075984\_contig\_number\_70SRS077127\_contig\_number\_14939SRS077730\_contig\_number\_2804SRS140492\_contig\_number\_24065SRS142542\_contig\_number\_18886SRS143342\_contig\_number\_9340SRS148511\_contig\_number\_17104SRS148817\_contig\_number\_9970SRS893172\_contig\_number\_5688SRS893187\_contig\_number\_11756SRS893230\_contig\_number\_7260
246787
species
